# Why, and how, mixed methods research is undertaken in health services research in England: a mixed methods study

**DOI:** 10.1186/1472-6963-7-85

**Published:** 2007-06-14

**Authors:** Alicia O'Cathain, Elizabeth Murphy, Jon Nicholl

**Affiliations:** 1 Medical Care Research Unit, School of Health and Related Research, University of Sheffield, Regent Street, Sheffield S1 4DA, UK; 2 School of Sociology and Social Policy, Law and Social Sciences Building, University of Nottingham, Nottingham NG7 2RD, UK

## Abstract

**Background:**

Recently, there has been a surge of international interest in combining qualitative and quantitative methods in a single study – often called mixed methods research. It is timely to consider why and how mixed methods research is used in health services research (HSR).

**Methods:**

Documentary analysis of proposals and reports of 75 mixed methods studies funded by a research commissioner of HSR in England between 1994 and 2004. Face-to-face semi-structured interviews with 20 researchers sampled from these studies.

**Results:**

18% (119/647) of HSR studies were classified as mixed methods research. In the documentation, comprehensiveness was the main driver for using mixed methods research, with researchers wanting to address a wider range of questions than quantitative methods alone would allow. Interviewees elaborated on this, identifying the need for qualitative research to engage with the complexity of health, health care interventions, and the environment in which studies took place. Motivations for adopting a mixed methods approach were not always based on the intrinsic value of mixed methods research for addressing the research question; they could be strategic, for example, to obtain funding. Mixed methods research was used in the context of evaluation, including randomised and non-randomised designs; survey and fieldwork exploratory studies; and instrument development. Studies drew on a limited number of methods – particularly surveys and individual interviews – but used methods in a wide range of roles.

**Conclusion:**

Mixed methods research is common in HSR in the UK. Its use is driven by pragmatism rather than principle, motivated by the perceived deficit of quantitative methods alone to address the complexity of research in health care, as well as other more strategic gains. Methods are combined in a range of contexts, yet the emerging methodological contributions from HSR to the field of mixed methods research are currently limited to the single context of combining qualitative methods and randomised controlled trials. Health services researchers could further contribute to the development of mixed methods research in the contexts of instrument development, survey and fieldwork, and non-randomised evaluations.

## Background

Both qualitative and quantitative methods can be used in the same study. This is variously called 'multi-method', 'mixed methods' or 'multiple methods' research [1], although there is a move to standardise terminology and use the label 'mixed methods research' for studies combining qualitative and quantitative methods [2]. There is an established body of knowledge about mixed methods research, discussing why this approach is used, how it can be used, and highlighting the challenges of using it in theory and in practice [3,4]. Recently, there has been an increased interest in mixed methods research in the fields of social and educational research both in the United Kingdom (UK) [5-9] and North America [10,11]. Over the last two years, journals devoted solely to mixed methods research have been launched – the Journal of Mixed Methods Research, and the International Journal of Multiple Research Approaches. Over the next two years at least half a dozen books on mixed methods research will be published by researchers in the UK, Europe and North America. Thus it is timely to consider why and how health services researchers use mixed methods research. This will help researchers to understand the relevance of the established and emerging body of knowledge on mixed methods research to their work. The aims of this paper are to describe why and how researchers undertake mixed methods studies in HSR and to consider whether researchers are exploiting the full range of justifications and types of approaches available to them.

Historically, health services researchers in the UK have used quantitative methodology. In the past decade or so they have welcomed qualitative methodology, detailing the contribution it can make to research in health care [12]. Health services researchers have also combined qualitative and quantitative research in a single study and contributed to a renewed interest in mixed methods research, by making the case for mixed methods research in HSR [13] and giving overviews of the key issues in mixed methods research in HSR [14-16]. This interest in mixed methods research in HSR has also occurred outside the UK [17].

The main contribution of the HSR community in the UK to the body of knowledge about mixed methods research has been in the use of qualitative research alongside randomised controlled trials (RCTs). Health services researchers have detailed the contribution of qualitative methods within a pilot RCT [18]; encouraged the use of mixed methods within an iterative phased approach to trials [19] and within contextual evaluations undertaken alongside trials [20]; used qualitative research to improve the design and conduct of a trial [21]; described the challenges of undertaking a process evaluation [22]; detailed the process of exploring apparent discrepancies between findings from the qualitative and quantitative components of a pilot trial [23]; and described how to integrate largely qualitative process evaluations with trial data and findings [24].

Mixed methods research has been discussed in the HSR literature, but there is little information about how commonly it is used, and why and how it is used in practice. When researchers in HSR have offered justifications for using a mixed methods approach, these have usually been related to the need for comprehensiveness. Researchers have pointed to the complexity of health care and the need for a range of methodologies to understand and evaluate these complexities [12,16,18,19,25]. There has also been a growing recognition of the importance of understanding the impact of the delivery and organisation of health services, with a focus on processes as well as outcomes, and the range of methodological approaches required to do this [26]. However, there are many justifications for using mixed methods research, apart from comprehensiveness, including increased confidence in findings, ensuring that disempowered groups in society are heard, and developing or facilitating one method by guiding the sampling, data collection or analysis of the other [14,27]. There are also many different ways of designing mixed methods studies. Many researchers have described the range of potential roles of qualitative and quantitative methods within a mixed methods study. We have displayed these in a pragmatic way, following the stages of research from development to dissemination [28] (see Table 1). The characteristics of mixed methods studies have been described in terms of the purpose of combining methods, the priority of methods within a study, and the sequence in which methods are used [29], with purposes including complementarity, confirmation, development [30]. In this paper we describe why and how researchers undertake mixed methods studies in HSR in the context of this body of literature.

**Table 1:** Roles of different methods within a mixed method study

STAGE	COMPONENTS	ROLES
Defining the research question		A qualitative method can generate a hypothesis for a quantitative method to test [38], establish the theoretical framework for the quantitative method [39], or help conceptualise the whole study [16].
Addressing the range of research questions	Understanding how interventions work in the real world	A complex intervention may operate differently in practice from the original intention and qualitative research can address how an intervention is used in practice while quantitative research is used to measure outcomes [40]. The strength of qualitative research to assess processes has been noted in social research [38].
	Getting a range of perspectives	Qualitative research can help researchers to gain access to the views of participants while quantitative research allows researchers to explore their own agenda [38].
Designing the study	Determining the sample	A quantitative method can facilitate the sampling strategy for a qualitative method [38], for example a survey can distinguish representative from non-representative cases [39].
	Improving the conduct of a method	When designing a trial, qualitative research may help to design appropriate recruitment strategies and information [21]. This could be used for other quantitative methods such as surveys.
	Designing study instruments	A qualitative method can help to design good survey instruments [16, 39, 41], and aid scale construction from them [38]. In the context of evaluation, it can identify the outcomes important to different stakeholders, for inclusion within instruments [32].
	Developing or optimising interventions	When evaluating an intervention like a service [42], qualitative methods can help to develop the intervention [18], develop an understanding of how the intervention works and who it might be most effective for [43], and indicate why the intervention has not worked [18].
Analysis		The results from one method can affect the analysis of the other method, or qualitative and quantitative *data *can be combined for further understanding [44]. For example, qualitative data can be 'quantitised', that is, numerically coded for analysis with the quantitative data.
Making use of the findings	Interpreting the findings	Each method can provide different aspects of a phenomenon [38]. A qualitative method can explain factors underlying relationships in a quantitative study [38], confirm or contradict survey findings, interpret statistical relationships, explore puzzling responses or results, or offer case study illustrations [39]. It may change the interpretation of findings [32], for example urging that a treatment is not rejected as ineffective simply because it was not used, but finding a way of it being used so that it might be effective [45]. In the context of evaluation, qualitative methods can describe the context in which the study operates, in particular what is going on with controls, thus aiding interpretation [32].
	Determining generalisability	A quantitative method can help to generalise a qualitative study [38], for example a survey can situate the context of case studies [1].
	Implementation	Qualitative methods can be used to consider the results of a study and their application within a real world context, drawing on pluralistic views of different stakeholders [32].

## Methods

### Design

A mixed methods design was used to explore the use of mixed methods research in HSR. A documentary analysis of the proposals, reports and publications from mixed methods studies in HSR was undertaken to explore how mixed methods studies were undertaken. Interviews were undertaken with researchers from these studies to explore why researchers undertook mixed methods studies in the way they did. The two methods were used sequentially, with the documentary analysis undertaken before the interviews. The two methods were given equal priority. In terms of the purposes of combining methods, methods were combined both for complementarity, where each method addressed a different aspect of the research question, and for development where the quantitative component facilitated sampling for the qualitative component [30].

### Documentary analysis

The Department of Health is a key commissioner of HSR in England and summaries of funded studies are listed on their website [31]. This website was used as the source of HSR studies for this research. All summaries of research studies listed under ten programmes of research on the Department of Health website were read by one of the authors (AOC). Studies using primary health research were included. Studies undertaken as single studies rather than as part of a programme, initiative or fellowship were included because detailed summaries were available for the former whereas summaries were not available or had no methodological detail for latter types of studies. Projects with a quantitative and a qualitative component were classified as mixed methods studies. Details of this are described elsewhere [27]. When a mixed methods study was identified, the lead researcher of each study was written to with a request for the research proposal, the final report for completed studies, and any emerging publications. Documentation was read by one researcher (AOC) and a number of issues around how the study had been undertaken were coded on a data extraction form. Issues included the methods used, roles of methods, priority and purpose of methods, approach taken to integration, quality of components, and types of publications emerging. The issues of relevance to this paper were whether a justification was given for using mixed methods and the roles of each method within a study. The data extraction form was structured, based on roles discussed in the literature. There was space for free-text comments for each structured item. For example, if a justification was given for using mixed methods research then this was written on the data extraction form.

### Interview study

The aim of the qualitative component of the study was to explore why researchers in HSR undertook studies in the way they did, and explore facilitators and barriers to this approach. Therefore it was relevant to interview researchers who had undertaken mixed methods studies in HSR. Researchers working on the studies included in the documentary analysis were sampled. Researchers' names were available from lists of applicants on proposals, lists of authors on reports, and lists of authors on articles. Purposive sampling was undertaken [32] to include a range of types of researchers – qualitative and quantitative researchers – and a range of types of mixed methods studies. An attempt was made to maximise variation within the sample by including research situated in different types of departments such as nursing, research units, primary care, and psychiatry; and research funded from different Department of Health programmes. Face-to-face semi-structured interviews were undertaken with researchers, focusing on their views of mixed methods studies generally and on the study included in the documentary analysis. Interviews lasted approximately one hour, were recorded, and transcribed verbatim.

### Analysis

For the documentary analysis, codes from the data extraction form were entered into SPSS. Percentages of studies in each category were calculated. Free-text comments were transcribed from the data extraction form into Word. Comments were read, themes were identified, and the frequency of each theme across the studies was counted. The interviews were analysed using the first stages of Framework [33]. Framework was chosen as a suitable approach because it allows the researcher to explore their agenda explicitly while also allowing other themes to emerge from the analysis. A thematic framework was developed based on the research question and familiarisation with the first few transcripts, and then applied to each transcript using the computerised qualitative software package WinMAX [34]. The data extracts for each theme or sub-theme were read and further coding was undertaken within them to organise and understand the data. Themes included paradigms, team working, quality of studies, justifications for using mixed methods and publishing studies. The theme of relevance to this article was the justifications given by researchers for undertaking mixed methods studies. When exploring justifications for using mixed methods research, the documents were used as the public face, and the interviews as the private face, of studies.

## Results

### Response

18% (119/647) of primary health research studies were classified as mixed methods research. Documentation was received for 81 studies and on reading the full documentation six studies were reclassified as not meeting the inclusion criteria, leaving 75 mixed methods studies. There were 45 proposals available for 75 studies (60%) and 48 reports available for 52 completed studies (92%) in the documentary analysis. Twenty-two researchers were approached for interview and 20 agreed to be interviewed.

### Incidence of mixed methods studies in HSR

The proportion of studies classified as mixed methods research increased over time from 17% of studies commissioned in the mid 1990s to 30% in the early 2000s (Table 2). The ability to classify a study accurately as mixed methods research depended on the level of detail available in the summaries for each study, and this varied between and within funding programmes. Perhaps the most interesting finding was that even prior to 1995 a considerable proportion of HSR studies funded through these programmes were mixed methods studies.

**Table 2:** Incidence of mixed methods studies commissioned by the Department of Health Research & Development programme 1994–2004

Programme	%	n	N
Current programmes			
Service Delivery and Organisation (SDO)	46%	28	61
Health Technology and Assessment (HTA)	9%	14	136
New and Emerging Applications of Technology (NEAT)	9%	2	21
Past programmes			
Maternal and child health	17%	8	48
CVD and stroke	8%	4	49
Implementation	36%	10	28
Primary secondary care interface	18%	11	62
Primary dental care	20%	8	41
Forensic mental health	17%	5	30
Policy Research Programme (PRP)	19%	29	157

Year			
Pre 1995	17%	33	191
1996–1998	15%	28	189
1999–2001	16%	25	156
2002–2004	30%	33	111

Total	18%	119	647

### Description of the 75 mixed methods studies and 20 interviewees

There was an even distribution of studies across funding programmes and year of funding, with the exception of one funding stream in which there was only one study (Table 3). The studies were mainly evaluations and combinations of survey and fieldwork, with a few feasibility and instrument development studies. As planned in the sampling strategy for the interviews, interviewees had been involved in a range of methodologies, types of studies, and programmes (Table 3).

**Table 3:** Description of 75 mixed methods studies in the documentary analysis and 20 interviewees

Characteristics of 75 studies	% (n)
**Funding programme**	
Current programmes	
SDO	27% (20)
HTA	17% (13)
NEAT	1% (1)
Past programmes	33% (25)
PRP	21% (16)

**Year of funding**	20% (15)
Pre 1995	20% (15)
1996–1998	33% (25)
1999–2001	17% (13)
2002–2004	29% (22)

**Type of study**	
Evaluation	46% (34)
*RCT*	*18% (14)*
*Other*	*28% (21)*
Feasibility study	7% (5)
*RCT*	*4% (3)*
*Other*	*3% (2)*
Fieldwork and survey	40% (30)
Instrument development	7% (5)

Characteristics of 20 interviewees	N = 20

**Type of researcher**	
Quantitative	11
Qualitative	9

**Type of study**	
Evaluation with RCT	6
Evaluation other	8
Survey and fieldwork	4
Instrument development	2

**Funding programme**	
Current programmes	
SDO	6
HTA	4
Past programmes	5
PRP	5

### Justifications for using a mixed methods approach

Justifications were offered in both documents and interviews, including those related to the intrinsic value and strategic purpose of using a mixed methods approach.

#### Intrinsic value of the approach

An explicit justification for using mixed methods research was given in only one third of proposals and reports in the documentary analysis (Table 4). The main justification was using different methods to address different questions or aspects of the overall research question so that the study was more comprehensive. The strengths and weaknesses of qualitative and quantitative methods were sometimes discussed as important in this context. Researchers made little or no use of justifications commonly discussed in the literature around increasing confidence in findings or the emancipation of marginalised groups. A small number of researchers did however discuss using qualitative research to bring a patient-centred approach to their study.

**Table 4:** Justifications given in documents for using mixed methods research

Justification	Proposals (N = 45)	Reports (N = 47)*
None	31	33
Comprehensiveness	13	12
Patient-centred	2	1
Confidence in findings	0	1
Good quality research	0	1

Adds to more than 100% due to two justifications given for one study *one report was a summary with insufficient detail for inclusion here

During the interviews, researchers' discussions of mixed methods research confirmed and elaborated some of the findings of the documentary analysis. Interviewees cited comprehensiveness as a driving force for the use of mixed methods research in HSR, wanting to address a range of questions and obtain a broader picture of a phenomenon. A comprehensive approach was seen as necessary due to the complexity of the issues under study, either the disease or the intervention, or the research environment in which the study was undertaken. The research environment was seen as particularly complex for policy-related research, with qualitative research undertaken to describe and understand a changing complex health service.

I'm not saying all the time, but a lot of the time in research it's difficult to get the whole picture without both. R12

It's less about the intellectual issue of mixed methods, it's more about the context in which those studies are conducted. And they kind, it seems to me they are necessary in areas of rapidly changing policy and practice. And because that's where they are conducted, they are inevitably complex and messy. R4

One argument for undertaking mixed methods studies was more prominent in the interviews than in the documentary analysis. Researchers associated qualitative research with gaining the views of patients. The patient voice was perceived as important in HSR because of its usefulness in understanding the complexity of a disease, an outcome, or an intervention, and grounding the research more in the real world. Service providers were also considered to be important voices for this reason.

Until you get down to hearing the actual experience, how people have described what the [intervention] was like for them and the problems they had with it, and the difficulties they had with it, as well as the positives, that you really get down to the nitty gritty, just what it is about this actual intervention that works and for who. R11

Thus the justifications for using a mixed methods approach were usually grounded in the applied nature of HSR, emanating from a need to engage with the real world and address policy related issues in a complex research environment, rather than any ideological stance. The desire to hear patient and provider voices was part of this applied and pragmatic approach rather than based on an ideology of emancipation of marginalised groups. Further, when discussing why they had taken a mixed methods approach, or why it was important to take such an approach, interviewees often justified the inclusion of a *qualitative *component within a study rather than justifying the use of a mixed methods approach. This may reflect the context of HSR in England as predominantly quantitative with an increasing acceptability and use of qualitative methods alongside quantitative methods. There was also a personal enthusiasm for mixed methods research amongst some of the researchers who felt that a justification was needed for why a study did not use a mixed methods approach. These researchers had been inspired to use mixed methods by other researchers or research projects earlier in their careers. Again, this enthusiasm was based on what researchers believed a mixed methods approach could deliver in the type of research field they worked in, rather than a belief in mixed methods research *per se*.

#### Using the approach for strategic purpose

Interviewees emphasised the need to undertake mixed methods research when the research questions demanded this approach.

it's got to be for the question. You can't just say 'we've all got to do mixed methods research'. It has to be 'what is the question, and which methods are the most appropriate ones to use in that circumstance', and that's got to be the driver. R17

This point was made as part of a concern that some funding bodies were 'into' mixed methods research, asking either explicitly or implicitly for this approach. These funding bodies were identified as pushing for the inclusion of qualitative research within totally quantitative studies and sometimes *vice versa*, thus forcing the existence of mixed methods studies. This 'push' was rarely explicit, where a funding body forced two sets of researchers to work together, but arose from researchers' perceptions that mixed methods research was required to obtain funding. This was discussed in the context of evaluative research, both randomised and non-randomised studies. Researchers feared that responding to funding bodies' desire for mixed methods studies could result in paying lip service to mixed methods research. Thus they shared a previously voiced concern in HSR that mixed methods studies may not be undertaken for their intrinsic value but more for a strategic purpose of obtaining funding [13].

So there is a sense in which in some studies you shoe horn a bit of qualitative work in because you think that will get you the funding. R7

AOC: Did [the funder] actually make a call for mixed methods?

I: I think they still use multi-disciplinary actually, which is not the same thing (laugh).

AOC: Right. That's how you read it when they say multi-disciplinary, you think...

I: That's now how I interpret it. R13

Obtaining funding was not the only strategic use of mixed methods research described by these interviewees. Other strategic uses discussed were undertaking qualitative research within a study as a safe guard against a null trial which might prove to be unpublishable without a qualitative component, and undertaking a survey within a study as a 'safety net' for dissemination because of the credibility it offered.

But sometimes it is handy to have that kind of safety net of, you know, a nice big survey behind your fieldwork. R10

Even if researchers did not discuss their own strategic uses of mixed methods research, they expressed concerns that the approach was a fashionable trend with a potentially detrimental effect on quality research.

This kind of mixed methods is a bit like apple pie, I mean, you know, people are thinking it's a good idea. The question is how it is done and for what purposes? R15

### Characteristics of mixed methods studies

In the documentary analysis, half of the mixed methods studies were evaluations and a further third used a combination of survey and fieldwork to understand an issue (Table 5). There were few examples of feasibility studies or instrument development studies. Two thirds of the studies were classified as explanatory rather than exploratory. The qualitative component of most studies was an interview study, with some use of case studies and focus group studies. Case studies often included focus groups, interviews, documentary analysis and observation so these methods were more frequently used than suggested in Table 5. Even so, there appeared to be heavy reliance on individual interviews within these studies. There was more variation in the quantitative component of studies, although evidence of reliance on surveys.

**Table 5:** Characteristics of the 75 mixed methods studies in HSR

Characteristic	Proposal N = 43* % (n)	Report N = 48% (n)
Type of study		
Evaluation	53% (23)	46% (22)
Fieldwork and survey	35% (15)	40% (19)
Feasibility study	7% (3)	10% (5)
Instrument development	5% (2)	4% (2)

Purpose of the study		
Explanatory	65% (28)	58% (28)
Exploratory	28% (12)	42% (20)
Both	7% (3)	0% (0)

Components		
Qualitative:	79% (34)	67% (32)
Interview study	79% (34)	67% (32)
Focus group study	12% (5)	23% (11)
Observation	2% (1)	10% (5)
Case studies	19% (8)	40% (19)
Documentary analysis	2% (1)	2% (1)
Other e.g. diaries	0% (0)	4% (2)
Quantitative:	40% (17)	62% (30)
Survey	40% (17)	62% (30)
Other observational study	26% (11)	19% (9)
RCT	28% (12)	21% (10)
Other intervention study	28% (12)	23% (11)
Economic	40% (17)	23% (11)
Other	7% (3)	2% (1)

* two studies were not mixed methods studies at the proposal stage and were not included here

### Roles of methods

The main roles of the quantitative components in these mixed methods studies were to describe a phenomenon, test the effectiveness of an intervention, and explain variability (Table 6). Roles specific to mixed methods studies were determining the sample for the qualitative component and generalising qualitative findings. This latter role rarely occurred in the mixed methods studies here. This lack of use of quantitative methods in this role may reflect the dominance of quantitative methods in HSR and the fact that the quantitative component is rarely in a supporting role to the qualitative component. Or this role may be used more in programmes of research rather than in single studies. Nevertheless it is a role that is not widely used and one which researchers may wish to consider for more use.

**Table 6:** Roles of methods in mixed methods studies in HSR

	Proposal (N = 43)	Report (N = 48)
**Role of quantitative**		
Test effectiveness	47% (20)	46% (22)
Describe	40% (17)	54% (26)
Explain variability	26% (11)	21% (10)
Determine sample for qualitative	35% (15)	40% (19)
Generalise the qualitative findings	5% (2)	4% (2)
Generate consensus	5% (2)	2% (1)
Psychometrically test	2% (1)	4% (2)
Provide topic guide for qualitative	2% (1)	4% (2)

**Role of qualitative**		
Develop the research question	0% (0)	0% (0)
Generate hypothesis	0% (0)	0% (0)
Establish theoretical framework	2% (1)	2% (1)
Determine sample	2% (1)	0% (0)
		
Generate content of instrument	30% (13)	10% (5)
Cognitively test instrument	9% (4)	6% (3)
Aid scale construction	0% (0)	2% (1)
Test validity of questionnaire	0% (0)	2% (1)
		
Develop intervention	16% (7)	13% (6)
Pilot intervention	2% (1)	2% (1)
Describe intervention	12% (5)	4% (2)
Study how intervention works	19% (8)	8% (4)
Study how the service works	5% (2)	13% (6)
Study intervention in practice	12% (5)	6% (3)
Process evaluation	14% (6)	4% (2)
Views of intervention	2% (1)	8% (4)
		
Determine outcomes and measures	0% (0)	0% (0)
Improve trial methodology	5% (2)	2% (1)
Explore RCT as social construct	2% (1)	0% (0)
Facilitate user involvement	0% (0)	2% (1)
		
Explore an issue	33% (14)	38% (18)
Uncover issues inaccessible to quant	7% (3)	0% (0)
Explore acceptability of care	7% (3)	6% (3)
Assess effectiveness	0% (0)	2% (1)
		
Explain relationships	12% (5)	10% (5)
Explore unusual findings	0% (0)	0% (0)
Explore issues from quantitative	7% (3)	4% (2)
Explore identified unusual groups	2% (1)	0% (0)
Offer case illustrations	5% (2)	6% (3)
Offer depth information on new cases	12% (5)	6% (3)
Confirm a quantitative finding	2% (1)	4% (2)
		
Understand results in real world	7% (3)	2% (1)

The main roles of the qualitative components were to explore an issue, and generate the content of a questionnaire or measurement tool, the latter being a role specific to mixed methods studies (Table 6). Qualitative research was also used to study a range of aspects of an intervention or service. However, there were some gaps in the roles taken by the qualitative research compared with roles identified in the literature (Table 1). They were rarely used to generate hypotheses for testing within a study, and again, this may reflect the fact that single studies rather than programmes were included here. Their role in questionnaire development was clearly focused on identifying the content of a questionnaire and less so on further development of the questionnaire, for example, with cognitive testing. They were not used to determine which outcomes to measure in a study, and again this may reflect the fact that programmes of research were not included here.

Components could have more than one role within a study. For example, a qualitative component might be used both to explore an issue in its own right as well as to develop the content of a questionnaire. That is, a component might have a stand alone role as well as a supportive role in relation to another method.

### Purposes and processes of combining methods

The main purposes of combining methods in studies in the documentary analysis were complementarity (methods used to address different aspects of the same question), expansion (methods used to address different questions), and development (one method used to inform the development of another) [30]. Confirmation, where the results of two methods converge, was rarely the purpose of mixing methods in these studies (Table 7). This reflects the justifications for using a mixing methods approach in the first place. The documentary analysis suggested that researchers either prioritised the quantitative component or gave the two components equal weighting. It was unusual to find studies where a qualitative component predominated. These were mainly case studies where a survey and analysis of routine data was undertaken alongside interviews, observation and documentary analysis. In two cases the qualitative dominance had not been planned but was a result of the failure of the quantitative component of the study. The quantitative dominance is not surprising given the history of HSR as drawing predominantly on quantitative methods, and perhaps it is surprising to find any studies with qualitative dominance. Nonetheless, one could argue that researchers could consider the relevance of qualitative dominant designs in HSR. A study could have a range of methods with three or four combinations of methods occurring within a study, some of which were sequential and some of which were concurrent. In two thirds of studies, methods were used concurrently (Table 7) and in a slightly lower proportion they were used sequentially.

**Table 7:** Purposes and processes of combining methods in 75 mixed methods studies in HSR

Characteristic	Proposal N = 43	Report N = 48
Purpose of combining methods		
Confirmation	2% (1)	6% (3)
Complementarity	60% (26)	40% (19)
Expansion	47% (20)	46% (22)
Development	44% (19)	35% (17)

Priority		
Mainly qualitative	5% (2)	10% (5)
Mainly quantitative	65% (28)	54% (26)
Equal	30% (13)	35% (17)

Sequence		
Sequential	65% (28)	54% (26)
Concurrent	70% (30)	69% (33)

## Discussion

One fifth of HSR studies funded by the Department of Health in England between 1994 and 2004 were mixed methods studies, with some evidence that this had increased over time. The frequency of use may have been different if fellowships and programmes of research had been included in the study. Evidence elsewhere around the frequency of use of mixed methods research is based on publications rather than funded studies and thus there is no evidence to support or refute this finding. Other research indicates both lower and higher use of a mixed methods approach in the health field. For example, only 1% (37/3830) of papers in clinical journals were found to use both qualitative and quantitative methods, although the focus of the study had been to identify qualitative research [35]; and 10 of 26 (38%) evaluations located in health journals in 1995 used a mixed methods approach [32]. In other research fields, it has been cited that 13% (145/1156) of articles in education journals used a mixed methods approach [5], and 8% (14/170) of primary research studies on long term conditions were mixed methods research [36]. Despite the lack of supporting evidence, it appears that mixed methods studies are common enough in HSR to be identified as an important methodological approach warranting further consideration. Thus established and emerging literature on mixed methods research is highly relevant to the HSR community.

Researchers justified the use of a mixed methods approach on pragmatic rather than ideological grounds – they worked in an applied field studying complex issues in complex environments. Qualitative research helped them to address and understand these complexities, and bring in the voices of users and providers of services to help them to do so. A pragmatic justification for using mixed methods research has been found recently among social researchers in the UK [8]. Thus this is not unique to the HSR community. Indeed although much of the established and emerging literature on combining qualitative and quantitative methods discusses the philosophical challenges of taking this approach, this literature also addresses a pragmatic stance [10,11], making it highly relevant to HSR. However, a potential downside of the driver of mixed methods research being the practical need to use a range of methods is that researchers may not view their research in the context of the body of learning about mixed methods research, thus limiting the knowledge they yield from this approach [27].

The main justification for using a mixed methods approach in HSR was comprehensiveness rather than the range of justifications discussed in the literature. In particular researchers did not use a justification of increased validity when different methods with different strengths offer convergence of findings. This lack of use of classic triangulation, or confirmation, in HSR is welcome because concerns have been expressed about the difficulties of using methods with a purpose of confirmation [13], particularly as an indicator of validity [37]. Having said this, confirmation is a key focus within much of the mixed methods literature and the HSR community will need to be aware of this when reading the literature about mixed methods research.

Researchers in HSR discussed the intrinsic value of mixed methods research but also discussed its use for strategic purpose. The main strategic use was to gain funding and researchers expressed concern about this because it might result in poor quality components of studies. Again, this was not confined to the HSR community because social researchers have also stressed the centrality of the research question in determining methods and expressed concerns that mixed methods research has become a fad because of funding bodies' desire for this approach [8].

Researchers in HSR drew on a range of both quantitative and qualitative methods but tended to make a lot of use of surveys and interviews. This frequency of use of interview studies in mixed methods research is very similar to social research where 71% of mixed methods articles were based on interview studies compared with 67% here [5]. Of course the questions posed in HSR may be best addressed by individual interviews, but this may also indicate a neglect of potentially useful qualitative methods such as observation and documentary analysis. Researchers appeared to be drawing on a wide range of roles for the different methods within studies in the contexts of evaluations, survey and fieldwork, and instrument development. A lack of use of some roles might be due to the fact that they are best suited for use within programmes of research rather than single studies but nonetheless researchers may wish to consider the range of roles of methods detailed here and the relevance of these roles within their future research. A lot of use was made of predominantly quantitative designs which again may be most suitable for HSR or may indicate a lack of variety of designs.

Perhaps the most interesting finding here is that a combination of qualitative research with a randomised controlled trial is a minority of the types of mixed methods studies undertaken in HSR yet this is where health services researchers are making a strong contribution to the literature on mixed methods research. This may not seem surprising given the significance of the randomised controlled trial within HSR. However, it is important that researchers in HSR note the frequency with which they combine methods in the context of non-randomised evaluations, survey and fieldwork studies, and instrument development, and recognise that they can make a contribution to the development of mixed methods research in these contexts too.

This paper is based on an empirical study of mixed methods research in a specific research field. It focuses on why and how this approach is undertaken within HSR. The empirical study covered a number of issues of importance to undertaking mixed methods research in HSR which are not reported here, including paradigm differences between researchers, the effect of team working on research outputs, assessment of the quality of mixed methods studies, and the facilitators and barriers to integration within studies. These will be reported in further papers.

### Limitations

The responses to requests for documentation were representative of the specified population of mixed methods studies. However, there was a lower response to requests for proposals than reports. Assessment of the study documentation did not include double coding to check for inter-rater or intra-rater reliability. The studies here did not include all mixed methods studies in HSR funded in England between 1994 and 2004. HSR was funded by Regional Health Authorities, charities, and research councils over that time period. In addition, programmes, initiatives and fellowships were not included. There is no reason to believe that HSR funded through other sources is different from the HSR funded by the Department of Health, except in terms of the extent to which it was policy related. However, the research cultures of different countries in North America and Europe may be different from England and the findings are likely not to be generalisable outside the UK. Transferability is relevant to the findings from the qualitative component [37]. The context of the qualitative research has been described to allow readers to consider the transferability of the findings to other settings. The context was researchers in HSR, mainly in England, where quantitative methodology has dominated historically.

## Conclusion

Mixed methods research is common in HSR and the recent surge of interest in this approach internationally is highly relevant to the HSR community. Mixed methods research is used in HSR on pragmatic rather than ideological grounds, to engage with the variety of questions relevant to the complexity of health care. However, there is also strategic use of mixed methods research to gain funding and credibility, and concerns that this may hinder the utility of this approach. A range of methods, roles of methods and designs have been used in HSR but researchers may wish to reflect on the range available for use and their utility for addressing the range of questions in HSR. The HSR community has made a strong contribution to methodological development in the context of combining randomised controlled trials and qualitative research. It is in a position to make a strong contribution to methodological development in mixed methods research beyond this in the context of non-randomised evaluation, survey and fieldwork, and instrument development, and may wish to take the opportunity to do so.

## Competing interests

The author(s) declare that they have no competing interests.

## Authors' contributions

AOC conceived the study, collected and analysed the data, and wrote the first draft of the paper. EM and JN contributed to the design and analysis of the study and commented on drafts of the paper. All authors read and approved the final manuscript.

## Pre-publication history

The pre-publication history for this paper can be accessed here:


